# The Role of Wildlife and Climate Change in the Emergence of One Health-Related Viral Diseases in Australia

**DOI:** 10.3390/v18091001

**Published:** 2026-09-11

**Authors:** Md. Wajed Ali, Md. Eram Hosen, Md. Mizanur Rahaman, Robert Kinobe, Martina Jelocnik, Subir Sarker

**Affiliations:** 1 Biomedical Sciences and Molecular Biology, College of Medicine and Dentistry, James Cook University, Townsville, QLD 4811, Australia; md.wajedali@my.jcu.edu.au (M.W.A.); mderam.hosen@my.jcu.edu.au (M.E.H.); mdmizanur.rahaman@my.jcu.edu.au (M.M.R.); 2 Australian Institute of Tropical Health and Medicine, James Cook University, Townsville, QLD 4811, Australia; 3 College of Science and Engineering, James Cook University, Townsville, QLD 4811, Australia; robert.kinobe@jcu.edu.au; 4 Centre for Bioinnovation, University of the Sunshine Coast, Sippy Downs, QLD 4556, Australia; mjelocni@usc.edu.au; 5 School of Science, Technology and Engineering, University of the Sunshine Coast, Sippy Downs, QLD 4556, Australia

**Keywords:** wildlife viruses, climate change, One Health, Australian wild animals, zoonotic

## Abstract

Wildlife-origin viral diseases are an increasing One Health challenge in Australia, with climate change emerging as a key driver of disease emergence, transmission, and spillover. Australian wildlife, particularly marsupials, birds, and bats, serve as important reservoirs of zoonotic and vector-borne viruses. However, the combined influence of wildlife reservoirs and climate change on viral disease ecology in Australia has not been comprehensively synthesised. This review examines the epidemiology, significance, and climate sensitivity of wildlife-associated viral diseases and evaluates how climate change and variability affect reservoir host ecology, vector biology, viral persistence, and host–pathogen–vector interactions. Thirteen major wildlife-associated viruses of One Health importance were identified, with transmission predominantly involving wildlife reservoirs and mosquito vectors. Temperature and rainfall were the principal climatic drivers influencing the transmission of Ross River virus (RRV), Barmah Forest virus (BFV), Murray Valley encephalitis virus (MVEV), Japanese encephalitis virus (JEV), West Nile virus/Kunjin virus (WNV/KUNV), and avian influenza A virus (AIV). For most mosquito-borne viruses (MBVs), temperature exhibited a non-linear relationship with transmission, while above-average rainfall generally increased outbreak risk by promoting mosquito abundance, habitat availability, and reservoir host activity. Significant knowledge gaps remain in wildlife virome surveillance, climate–disease pathways, and integrated One Health surveillance. Strengthening climate-informed surveillance that integrates wildlife, vectors, genomics, epidemiology, and predictive modelling will improve outbreak preparedness and reduce the risk of viral emergence and spillover in Australia.

## 1. Introduction

Wildlife-origin viral diseases are the predominant source of emerging infectious diseases (EIDs) affecting humans and animals and represent a growing global One Health challenge. Approximately 60% of EID events in humans are zoonotic, and 70–72% of these originate from wildlife reservoirs [1]. Over recent decades, the emergence of novel viral diseases from wildlife populations has increased, highlighting the importance of understanding the ecological drivers of pathogen emergence [2,3]. Wildlife species play critical roles in maintaining viral diversity and persistence, often without clinical disease. Bats, birds, rodents, marsupials, and non-human primates are recognised as important reservoirs of zoonotic viruses, including RRV, BFV, JEV, AIVs, and Hendra virus (HeV) [4,5,6]. Although an estimated 10,000 viral species may be capable of infecting humans, most remain undiscovered in wildlife populations [7,8].

Climate change is increasingly recognised as a major driver of EIDs because it alters ecological interactions among pathogens, reservoir hosts, vectors, domestic animals, and humans [7,9,10]. Rising temperatures, changing rainfall patterns, and increasing frequency of extreme weather events influence vector abundance and competence, reservoir host ecology, environmental pathogen persistence, and opportunities for disease transmission [9,10,11,12,13]. These climate-driven changes can modify host–pathogen–vector interactions, alter disease distributions, and increase the likelihood of zoonotic spillover. However, the magnitude and direction of these effects vary across pathogens, ecosystems, and geographic regions.

Australia provides a unique setting for examining climate-sensitive viral diseases because of its distinctive wildlife fauna and environmental conditions. Marsupials, birds, and bats serve as natural reservoirs or amplifying hosts for numerous zoonotic and vector-borne viruses of One Health importance [14,15]. At the same time, Australia is experiencing increasing climatic variability, environmental change, and expanding human–wildlife interfaces, all of which may influence disease emergence and transmission [7,13,14]. The country’s tropical and subtropical regions also support abundant mosquito populations, particularly *Aedes* and *Culex* species, facilitating the transmission of several endemic and emerging arboviruses [16]. Despite growing evidence linking climate change and variability to disease emergence, understanding of the relationships among climate variability, wildlife reservoir ecology, viral transmission, and spillover risk remains fragmented. This knowledge gap is particularly relevant given the recent increase in mosquito-borne viral diseases in Australia, including substantial rises in RRV notifications during 2023–2024 [17]. In addition, wildlife virome surveillance remains limited, constraining the detection of emerging viral threats and understanding of climate-sensitive transmission pathways.

This review synthesises current knowledge on major wildlife-associated viral diseases of One Health significance in Australia, including mosquito-borne viruses (MBVs), bat-associated viruses, and avian influenza A viruses (AIV). It examines their epidemiology, reservoir hosts, transmission pathways, and significance for human and animal health, and evaluates how climate change and variability influence reservoir host ecology, vector biology, viral persistence, and determinants of spillover risk. Finally, the review identifies key knowledge gaps and future priorities for wildlife surveillance, climate-informed risk prediction, and One Health approaches to emerging viral disease preparedness and control in Australia.

## 2. Wildlife-Origin Viruses of One Health Importance in Australia

Wildlife-associated viral diseases represent a major component of EIDs affecting human and animal health, biodiversity, and agricultural systems worldwide [18,19]. The emergence of these pathogens is frequently associated with ecological, demographic, and environmental changes that increase interactions among wildlife, domestic animals, vectors, and humans [20,21]. In Australia, several notable zoonotic viruses, including HeV and Australian bat lyssavirus (ABLV), emerged locally from wildlife reservoirs rather than through international introduction [22]. Over the past four decades, wildlife-origin infectious diseases have increased substantially, highlighting the importance of wildlife surveillance and One Health approaches to disease prevention and control [20]. Major wildlife-associated viruses of One Health significance in Australia are summarized below and in Table 1.

### 2.1. Ross River Virus

**Figure 1:**
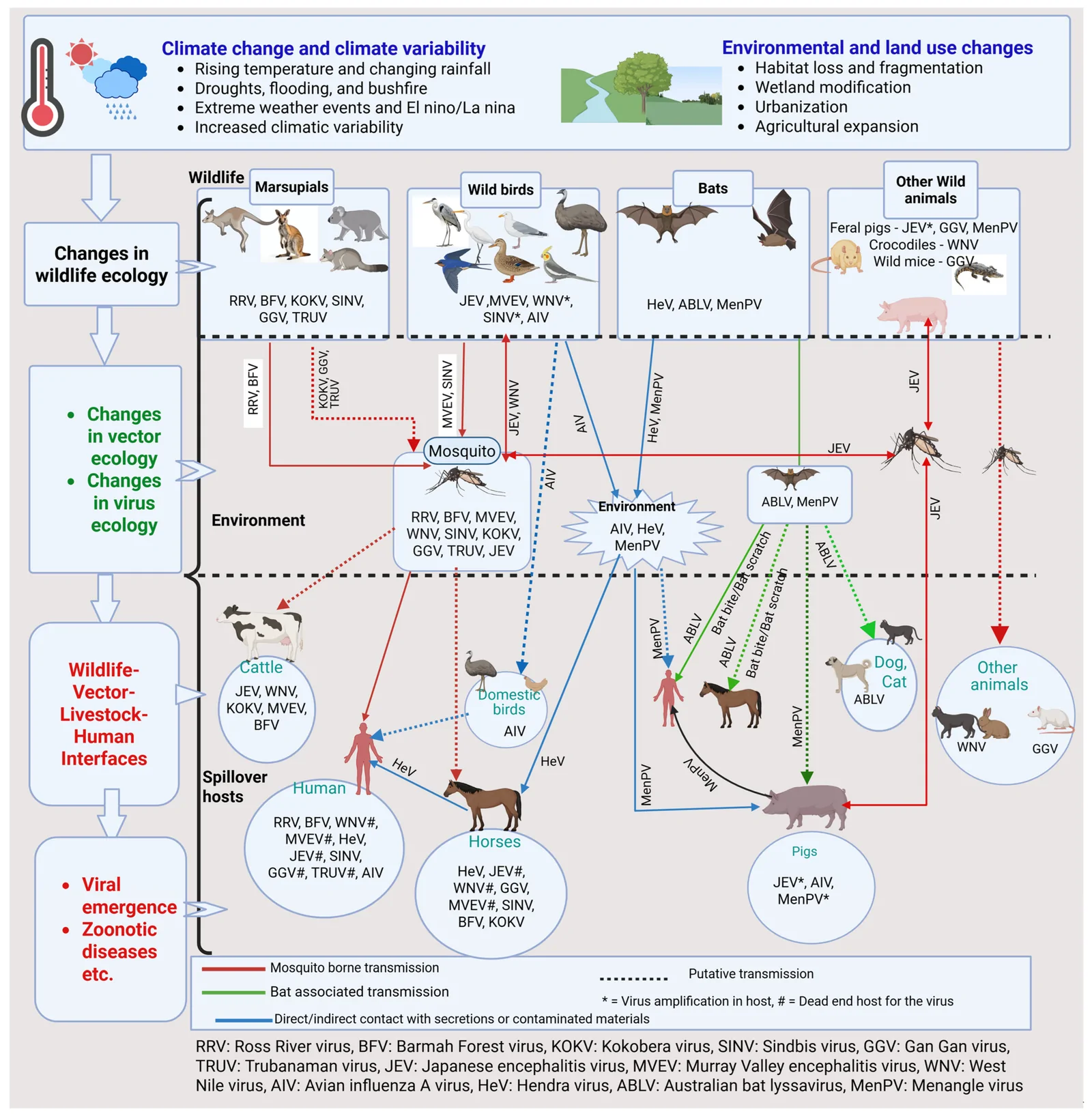
Conceptual framework linking climatic drivers for the emergence and spillover of key wildlife-origin One Health-related viruses. This figure illustrates the virus transmission dynamics between reservoir/maintenance host, vector and spillover host. Created in BioRender. Rahaman, M. M. (2026) https://BioRender.com/d5dz2qr.

RRV is the most frequently reported arboviral disease in Australia and imposes a substantial public health and economic burden across all states and territories [23]. National mosquito-borne disease notifications nearly doubled between 2023 and 2024, largely driven by increased RRV activity in Queensland, New South Wales, Victoria, and Western Australia [24]. RRV is an alphavirus within the family *Togaviridae* and is endemic throughout Australia, Papua New Guinea, and several Pacific Island nations [25,26]. Marsupials, particularly kangaroos and wallabies, are considered the principal reservoir hosts, although possums, birds, horses, and flying foxes may also contribute to virus maintenance (Figure 1 and Table 1) [23,27,28,29]. Transmission occurs through numerous mosquito species, with *Aedes vigilax*, *Aedes camptorhynchus*, *Culex annulirostris* and *Aedes notoscriptus* considered the primary vectors responsible for the majority of the previously identified RRV transmission events [30,31,32]. Human infection is typically characterised by polyarthritis, skin rash, fever, and myalgia, with outbreaks occurring across a wide range of ecological settings [27,33]. The broad host range and widespread distribution of competent mosquito vectors contribute to the continued public health significance of RRV in Australia.

### 2.2. Barmah Forest Virus

BFV is the second most common mosquito-borne alphavirus infection in Australia, causing approximately 1000 human cases annually [34,35]. Since its first isolation from *C. annulirostris* mosquitoes in Victoria in 1974, BFV has been detected throughout much of Australia and in Papua New Guinea [36,37]. A diverse range of wildlife and domestic animals have been implicated as potential reservoir hosts, including kangaroos, possums, koalas, quokkas, horses, cattle, dogs, and cats (Table 1) [38,39,40,41,42]. Human disease is clinically similar to RRV infection and is characterised by polyarthritis, fever, rash, and myalgia [43]. Transmission involves multiple mosquito vectors, particularly *C. annulirostris*, *A. vigilax*, and *A. camptorhynchus* [44,45]. The ecology of BFV transmission is strongly influenced by interactions among climatic conditions, vector abundance, wildlife reservoir hosts, and human exposure patterns (Figure 1) [46].

**Table 1:** Major wildlife- associated viruses of One Health importance in Australia: wildlife reservoirs, geographic distribution, transmission pathways/vectors, spillover risk, and detection methods.

Virus	Family	Genus	Species	Genome (Organization)	Reservoir/Host in Wildlife	Geographic Distribution	Transmission Pathways/ Vectors	Spillover Risk/Species Affected	Detection Methods	References
Ross River virus (RRV)	*Togaviridae*	*Alphavirus*	*Alphavirus rossriver*	Enveloped, (+) ssRNA	-Marsupials (western grey kangaroos, bandicoots, wallaroos, wallabies, common brushtail possums), -Australian birds (little corella, magpie larks, masked finch), wild eutherian mammals (rodents, bats)	Australia; Pacific Islands: Fiji	Mosquito-borne	Humans, horses (asymptomatic in cattle, dogs, cats)	Serology, microneutralization assays, RT-PCR, RT-qPCR	[6,29,39,41,47,48,49,50]
Barmah Forest virus (BFV)	*Togaviridae*	*Alphavirus*	*Alphavirus barmah*	Enveloped, (+) ssRNA	Marsupials (eastern grey kangaroos, brushtail possums, koalas, quokkas), wild eutherian mammals (rats)	Australia and Papua New Guinea	Mosquito-borne	Humans (asymptomatic in domestic mammals: cats, dogs, cattle, horses)	Serology	[38,39,40,41,42]
Japanese encephalitis virus (JEV)	*Flaviviridae*	*Orthoflavivirus*	*Orthoflavivirus japonicum*	Enveloped, (+) ssRNA	Ardeid birds (herons, egrets), feral pigs, meerkats	Australia (Torres Strait, Northern Australia), South-East Asia and the Western Pacific region: Thailand, China	Mosquito-borne	Humans, other birds (pigeons, sparrows, ducks, chickens), pigs (amplifying host), horses, cattle	Serology, PCR	[51,52,53,54,55,56]
Murray Valley encephalitis virus (MVEV)	*Flaviviridae*	*Orthoflavivirus*	*Orthoflavivirus murrayense*	Enveloped, (+) ssRNA	Australian birds (galahs, sulphur-crested cockatoos), waterbirds (herons), marsupials (eastern grey kangaroos, western grey kangaroos, agile wallabies), wild mice	Australia (Western Australia, Northern Territory, New South Wales, and Victoria), Papua New Guinea	Mosquito-borne	Humans, pigs, cattle, dogs, horses (occasionally)	RT-PCR, IgM ELISA	[57,58,59,60,61]
West Nile virus (WNV)	*Flaviviridae*	*Orthoflavivirus*	*Orthoflavivirus nilense*	Enveloped, (+) ssRNA	Wild birds, ardeid birds, white ibis, saltwater crocodiles, raptor birds, geese, marsupials (kangaroos, wallabies) wild eutherian mammals (eastern cottontail rabbits)	Australia, Africa, Asia, America, Middle East	Mosquito (vector) Wildlife–human interface	Humans, horses, cats, rabbits, cattle, chicken	RT-PCR, IgM ELISA	[50,55,62,63,64,65,66,67,68]
Avian influenza A virus (AIV)	*Orthomyxoviridae*	*Alphainfluenzavirus*	*Alphainfluenzavirus influenzae*	Enveloped, segmented, (–) ssRNA	Wild waterfowl (duck, geese, swan), migratory birds (gulls, terns, shorebirds), wild mammals, bats	Australia, Asia, Africa, America	Wild birds (Wildlife–livestock–human)	Humans, birds, domestic poultry, swine, horses, cattle, dogs and cats.	RT-PCR, HA/HI assays	[5,69,70,71,72,73,74,75]
Hendra virus (HeV)	*Paramyxoviridae*	*Henipavirus*	*Henipavirus hendraense*	Enveloped, (–) ssRNA	Flying foxes/fruit bat,	Australia (Queensland and New South Wales), Papua New Guinea	Bat–horse–human transmission	Humans, horses, pigs,	RT-PCR, serology	[76,77,78]
Australian bat lyssavirus (ABLV)	*Rhabdoviridae*	*Lyssavirus*	*Lyssavirus australis*	Enveloped, (–) ssRNA	Flying foxes, microbats	Australia	Bat spillover to human/horses Bat bites or scratches	Humans, horses,	RT-PCR, DFA, FAT, PCR	[79,80,81]
Sindbis virus (SINV)	*Togaviridae*	*Alphavirus*	*Alphavirus sindbis*	Enveloped, (+) ssRNA	Marsupials, feral rabbits, rodents, Shrews, treeshrews, emus, other birds, bats	Australia, China, Africa, Europe, and Asia	Mosquito-borne	Humans, native cats, rabbits (asymptomatic in horses, and emus)	ELISA, VNT, RT-PCR, qRT-PCR	[42,82,83,84,85,86]
Menangle virus (MenPV)	*Paramyxoviridae*	*Pararubulavirus*	*Pararubulavirus menangleense*	Enveloped, (–) ssRNA	Flying foxes	Australia	Bats-pigs-humans	Pigs, humans,	Serology (VNT), qRT-PCR	[87,88,89]
Kokobera virus (KOKV)	*Flaviviridae*	*Orthoflavivirus*	*Orthoflavivirus kokoberaorum*	Enveloped, (+) ssRNA	Marsupials (kangaroos, wallabies)	Australia (WA, NSW, QLD), Papua New Guinea	Mosquito-borne	Humans, horses, (asymptomatic in cattle)	HI, VNT, ELISA, RT-PCR	[49,58,65,90,91]
Gan Gan virus (GGV) and Trubanaman virus (TRUV)	*Peribunyaviridae*	*Orthobunyavirus*	*Orthobunyavirus ganganense* (GGV), *Orthobunyavirus buffaloense* (TRUV)	Enveloped, (–) ssRNA	-GGV Marsupials (kangaroos, wallabies, bush rats) -TRUV Western grey kangaroos, wallabies, feral pigs, foxes, quokkas	Australia (Queensland, New South Wales, and Western Australia), Papua New Guinea	Mosquitoes	-GGV Humans (asymptomatic in sheep, horses, cattle) -TRUV Horses, cows, rabbits,	Serology (neutralization test), RT-PCR, and genome sequencing	[40,42,91,92,93]

PCR: Polymerase Chain Reaction; RT-PCR: Reverse Transcription Polymerase Chain Reaction; qRT-PCR: Quantitative (Real-Time) Reverse Transcription Polymerase Chain Reaction; ELISA: Enzyme-Linked Immunosorbent Assay; IgM ELISA: Immunoglobulin M Enzyme-Linked Immunosorbent Assay; HA/HI: Haemagglutination and Haemagglutination Inhibition Tests; DFA: Direct Fluorescent Antibody Test; FAT: Fluorescent Antibody Test; VNT: Virus Neutralisation Test.

### 2.3. Japanese Encephalitis Virus

JEV is a mosquito-borne flavivirus and the leading cause of viral encephalitis across Asia and parts of the Western Pacific (Table 1) [56,94,95,96]. The virus was first detected in Australia during a 1995 outbreak in the Torres Strait and has since emerged as a significant zoonotic and veterinary concern [54]. JEV is maintained in enzootic transmission cycles involving ardeid waterbirds as reservoir hosts and pigs as amplifying hosts (Figure 1) [54,55,97,98,99,100]. In Australia, waterbirds, feral pigs, domestic pigs, and potentially flying foxes are thought to contribute to virus dispersal and maintenance [51,56,95,101]. Humans and horses act as incidental dead-end hosts but may develop severe neurological disease [99,102]. Transmission occurs primarily through *Culex* mosquitoes, with *C. annulirostris* considered the principal Australian vector [103,104]. The re-emergence of JEV in southeastern Australia during 2022 highlighted the capacity of climatic and ecological changes to facilitate the expansion of vector-borne zoonotic diseases into previously low-risk regions [101,105]. The interplay between these epidemiological factors is illustrated in Figure 1.

### 2.4. Murray Valley Encephalitis Virus

MVEV is a mosquito-borne *Orthoflavivirus* and one of Australia’s most important causes of arboviral encephalitis [106]. Endemic transmission occurs primarily in northern Australia, particularly in the Northern Territory and northern Western Australia, with periodic spread into southeastern regions causing sporadic outbreaks [107,108,109,110,111]. Waterbirds, especially ardeids, are considered the principal reservoir hosts, while *C. annulirostris* is the dominant mosquito vector (Figure 1 and Table 1) [60,111]. Most infections are asymptomatic or mild; however, severe neurological disease can occur in humans and horses [112,113]. Humans and horses are considered dead-end hosts because viraemia is insufficient to support onward transmission [114]. Recent human cases in Victoria and New South Wales demonstrate the continued public health importance of this climate-sensitive arbovirus [110].

### 2.5. West Nile Virus

WNV is a mosquito-borne *Orthoflavivirus* of global public and animal health importance. In Australia, the endemic Kunjin strain (WNV/KUNV) circulates primarily in northern regions, with periodic expansion into southern Australia following heavy rainfall and increased mosquito activity [115,116]. A notable outbreak occurred in 2011 when a virulent variant, designated WNVNSW2011, caused widespread neurological disease in horses in New South Wales [64]. Waterbirds, particularly herons and egrets, serve as the principal reservoir and amplifying hosts, facilitating virus maintenance and dispersal across Australia (Table 1) [55,60,117]. Transmission occurs predominantly through *C. annulirostris* mosquitoes, although other mosquito species may contribute locally (Figure 1) [67,118,119]. Humans and horses are incidental dead-end hosts because viraemia is insufficient to support onward transmission [120,121]. While most infections are asymptomatic or mild, severe neuroinvasive disease, including encephalitis, meningitis, and paralysis, can occur in both humans and horses [116,122,123]. The emergence of WNV/KUNV outbreaks following major rainfall and flooding events highlights the close interaction between climate variability, vector ecology, wildlife reservoirs, and disease emergence in Australia [64,115].

### 2.6. Avian Influenza A Virus

AIV is a globally distributed zoonotic virus within the family *Orthomyxoviridae* and is of major significance to human and animal health [124]. Wild waterfowl, seabirds, and migratory shorebirds constitute the primary natural reservoirs and play a critical role in viral maintenance, evolution, and global dissemination (Table 1) [5,69,125,126]. AIVs occur worldwide, including throughout Australia and Antarctic ecosystems [127,128]. Australia periodically receives AIV lineages through migratory shorebirds connecting Eurasian and North American flyways [124]. Most AIV cause mild infections in wild birds and poultry; however, low-pathogenic H5 and H7 strains can evolve into highly pathogenic avian influenza (HPAI) variants after circulation in domestic poultry populations [126,129,130]. Transmission to poultry generally occurs through direct or indirect contact with infected wild birds, contaminated water, feed, equipment, or fomites (Figure 1) [72,131]. Beyond birds, certain AIVs infect a range of mammalian hosts, including pigs, cattle, horses, dogs, and cats, highlighting their capacity for interspecies transmission and genetic adaptation [71,132,133,134]. The extensive movement of migratory birds and the virus’s propensity for mutation and reassortment make AIV a continuing One Health concern in Australia and globally [124].

### 2.7. Hendra Virus

HeV is a highly pathogenic zoonotic henipavirus endemic to Australia and represents one of the country’s most significant bat-borne disease threats [76,77]. First identified in 1994 during an outbreak in Brisbane, Queensland, HeV caused severe disease in horses and humans and remains an important veterinary and public health concern [76,78]. Flying foxes (*Pteropus* spp.) are the natural reservoir hosts, with black flying foxes (*P. alecto*) and spectacled flying foxes (*P. conspicillatus*) considered the primary reservoir species (Table 1) [77,135]. The virus spills over to horses through contact with bat urine, saliva, or contaminated feed and water sources, while transmission to humans occurs through close contact with infected horses (Figure 1) [136,137,138,139]. To date, all confirmed human infections have been associated with exposure to infected horses and have been characterised by a high case-fatality rate [140]. HeV infection in horses commonly causes severe respiratory and neurological disease, making horses important amplifying hosts for human infection. Spillover events occur primarily in Queensland and New South Wales and have been closely linked to environmental and ecological factors influencing flying fox ecology and behaviour [77,141]. As a result, HeV is widely regarded as a model example of wildlife-associated spillover at the human–animal–environment interface.

### 2.8. Australian Bat Lyssavirus

ABLV is an endemic zoonotic lyssavirus closely related to rabies virus and is capable of causing fatal encephalitic disease in humans and other mammals [142,143]. First identified in 1996, ABLV is maintained in Australian bat populations, including both flying foxes and insectivorous microbats [144,145]. Reservoir hosts include black flying foxes (*Pteropus alecto*), little red flying foxes (*P. scapulatus*), grey-headed flying foxes (*P. poliocephalus*), spectacled flying foxes (*P. conspicillatus*), and the yellow-bellied sheath-tailed bat (*Saccolaimus flaviventris*) (Table 1) [142]. Humans and horses are considered incidental hosts [81,146]. Transmission occurs primarily through bites, scratches, or exposure to saliva from infected bats (Figure 1) [147]. Clinical disease in humans is characterised by acute progressive encephalomyelitis and is almost invariably fatal without timely post-exposure intervention [81,146]. Although infection prevalence is low in wild bat populations, ABLV remains a significant public health concern because of its severe clinical outcome and widespread distribution among Australian bats [148,149].

### 2.9. Sindbis Virus

SINV is a mosquito-borne alphavirus widely distributed across Europe, Africa, Asia, and Australasia, including Australia and Papua New Guinea [84,150,151,152]. Although commonly isolated from Australian mosquitoes, human disease is reported infrequently [153,154]. Migratory birds are regarded as the principal amplifying hosts and likely contribute to the long-distance movement of the virus between continents [84,155,156]. Additional vertebrate hosts in Australia include marsupials, emus, rabbits, and horses (Table 1) [86,155]. The virus has been detected in multiple mosquito species, including *C. annulirostris*, *Aedes normanensis*, *A. camptorhynchus*, and *A. pseudonormanensis* (Figure 1) [157]. Human infection is associated with fever, arthralgia, myalgia, and rash, resembling other arthritogenic alphaviruses [84,158]. While clinical cases are uncommon in Australia, widespread serological evidence suggests that subclinical infection may be more common than currently recognized [84,159]. The involvement of migratory birds and multiple mosquito species highlights the ecological complexity of SINV maintenance and transmission.

### 2.10. Menangle Virus

MenPV is a bat-associated pararubulavirus first identified during a disease outbreak in pigs at Menangle, New South Wales, in 1997 [160,161]. The outbreak was associated with reproductive failure in pigs and influenza-like illness in two exposed workers, establishing the zoonotic potential of the virus [160,162]. Flying foxes, particularly black flying foxes (*P. alecto*), are recognized as the natural reservoir hosts (Table 1) [88]. Virus isolation from flying fox urine has confirmed the role of bats in maintenance and transmission (Figure 1) [88]. Pigs act as amplifying hosts and may develop reproductive abnormalities, stillbirths, and neurological disease [162,163]. Transmission is believed to occur through direct or indirect exposure to infectious excretions, including urine and faeces, although the exact transmission pathways remain incompletely understood [87,89,163]. MenPV highlights the importance of bat-associated paramyxoviruses as potential emerging zoonotic threats at the wildlife–livestock interface.

### 2.11. Kokobera Virus

KOKV is an endemic mosquito-borne *Orthoflavivirus* widely distributed across Australia and Papua New Guinea [164]. First isolated from *C. annulirostris* mosquitoes in Queensland in 1960, it belongs to a group of closely related orthoflaviviruses unique to Australia [159,164,165]. Marsupials, particularly kangaroos and wallabies, are considered important reservoir hosts, although antibodies have also been detected in horses and cattle (Table 1) [49,58,164,166]. The virus is transmitted by multiple mosquito species, including *Aedes vigilax*, *Aedes procax*, *Aedes notoscriptus*, and *C. annulirostris* (Figure 1) [167,168]. Human infection is uncommon but may cause febrile illness accompanied by polyarthritis, with occasional reports of neurological complications including encephalitis and myelitis [169,170,171]. The broad geographic distribution of mosquito vectors and wildlife reservoir hosts suggests that KOKV remains an under-recognized contributor to Australia’s arboviral disease landscape.

### 2.12. Gan Gan Virus and Trubanaman Virus

GGV and TRUV are mosquito-borne orthobunyaviruses reported in Australia and Papua New Guinea [93,172]. Serological evidence indicates widespread circulation among wildlife and domestic animals, including kangaroos, wallabies, cattle, horses, rabbits, feral pigs, and red foxes (Table 1) [40,42]. Macropods are regarded as the principal vertebrate hosts for both viruses [42]. GGV and TRUV have been isolated from a range of mosquito species, including *A. vigilax*, *C. annulirostris*, *Anopheles annulipes*, and *Anopheles meraukensis* (Figure 1) [93,173,174,175,176]. Humans are considered incidental dead-end hosts [177]. GGV has been associated with polyarthritis-like illness in humans, while TRUV is suspected to cause a similar clinical syndrome [178]. Despite evidence of circulation in wildlife and mosquitoes across several Australian states, the epidemiology and public health significance of these viruses remain poorly understood. Further surveillance is required to clarify their distribution, transmission ecology, and disease burden in Australia [92,93].

## 3. Effects of Climate Change and Variability on One Health-Related Viruses in Australia

Climate change and variability are increasingly recognised as major drivers of viral disease emergence because it alters the ecological processes that govern interactions among pathogens, wildlife reservoirs, vectors, domestic animals, and humans [179]. Changes in temperature, rainfall, humidity, wind patterns, and solar radiation can influence the survival, reproduction, distribution, and transmission of pathogens and their hosts [180]. In this context, climate variability denotes short-term, seasonal, and interannual fluctuations in climatic conditions, while climate change refers to long-term alterations in average climatic conditions, their variability, and the frequency or intensity of climatic extremes. Anthropogenic activities driving climate change have also increased the frequency and intensity of extreme weather events, including flooding, droughts, bushfires, and heatwaves, resulting in widespread ecological disruption that can modify disease transmission pathways and spillover risk [181].

**Figure 2:**
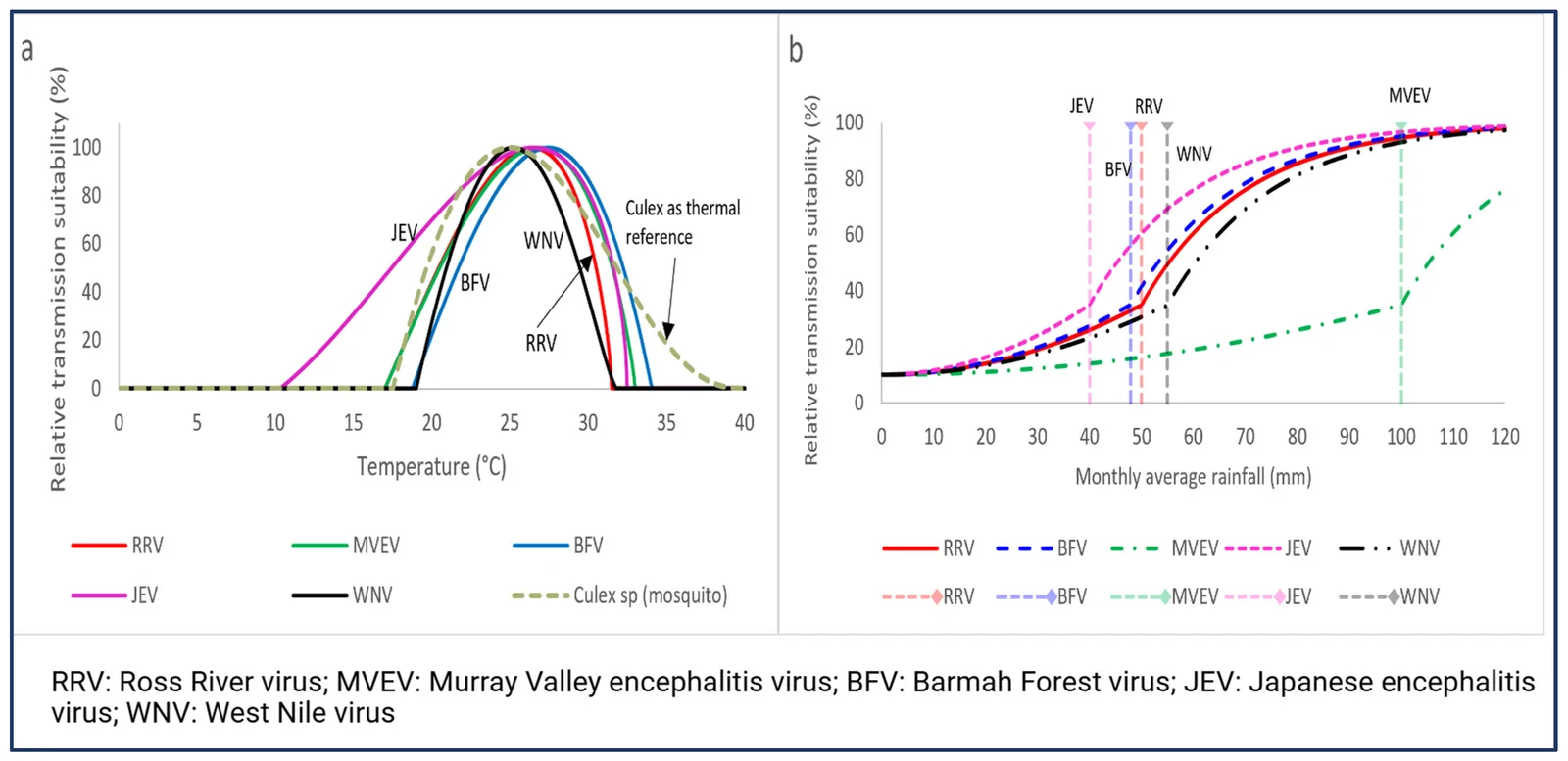
Conceptual temperature- and rainfall-dependent relative transmission suitability of five MBVs in Australia. Curves are normalized to a relative transmission suitability scale (0–100%) based on reported climatic thresholds, optimum ranges, direction of effect, and outbreak/case associations. The curves represent relative suitability rather than raw case counts. (**a**) Nonlinear effect of temperature on relative transmission suitability of selected viruses. A *Culex* mosquito thermal-suitability curve is included as a reference to illustrate the general unimodal temperature-dependent pattern of mosquito-associated transmission and is not a virus-specific curve. (**b**). Vertical dashed lines represent the average monthly rainfall thresholds identified in epidemiological studies (JEV = 40 mm, BFV = 47.9 mm, RRV = 50 mm, WNV/KUN = 55 mm, and MVEV = 100 mm). The curves are normalized (0–100%) to demonstrate the relative increase in transmission suitability above these rainfall thresholds. These curves are conceptual and serve to compare rainfall sensitivity, not to predict absolute transmission risk (see Table 2 and Supplementary Table S1 for data source; Figure created in Microsoft Excel and BioRender. Rahaman, M. M. (2026) https://BioRender.com/607hbr9).

The evidence synthesised in this review indicates that climatic drivers influence viral emergence through interconnected effects on reservoir host ecology, vector biology, viral persistence, environmental suitability, and host–pathogen–vector interactions. In this review, viral emergence broadly refers to the occurrence or increasing spread of a virus in a new or expanding host population, encompassing spillover into novel hosts and, when onward transmission is sustained, the establishment of a new transmission cycle or outbreak. Among the climatic drivers, temperature and rainfall are the most important determinants of transmission dynamics for major wildlife-associated viruses in Australia, including RRV, MVEV, BFV, JEV, WNV, and AIV (Table 2). However, the magnitude and direction of these effects vary among viruses, ecological systems, and geographical regions. For MBVs, transmission suitability generally exhibits a non-linear response to temperature, characterised by lower and upper thermal limits and an intermediate optimum range that reflects mosquito thermal biology and viral replication dynamics (Figure 2a). In contrast, above-average rainfall and flooding typically enhance transmission by increasing mosquito breeding habitats, vector abundance, and interactions among wildlife reservoirs, livestock, and humans. The rainfall thresholds associated with elevated transmission risk differ among viruses and regions (Figure 2b). The following sections examine how climate change and variability influence reservoir host ecology, viral evolution and persistence, vector biology, and host–pathogen–vector interfaces that collectively shape viral emergence and spillover risk in Australia.

### 3.1. Climate Change and Variability Effects on Wildlife Reservoir Host Ecology, Distribution, and Range Shifts

Climate change and variability are reshaping the distribution, abundance, behaviour, and interspecific interactions of wildlife reservoirs, thereby influencing viral transmission dynamics and zoonotic spillover risk worldwide. National climate projections based on Australia-wide CMIP6 projections indicate that Australia will continue to warm, with more extremely hot days and fewer extremely cool days; mean temperatures over the next 20 years are virtually certain to exceed those of the preceding 20 years. Projected rainfall changes are expected to remain spatially and seasonally heterogeneous. Climate models project declining cool-season rainfall across much of southern Australia, whereas the direction and magnitude of rainfall changes in northern and eastern regions remain more uncertain and vary by location, season, and emissions scenario [202].

These projected climatic changes provide an important context for understanding shifts in wildlife ecology. Changes in temperature and rainfall can influence food and water availability, habitat suitability, reproduction, survival, movement, and population distribution, thereby modifying wildlife–vector and wildlife–host interactions. Climate-driven changes in temperature, precipitation, habitat suitability and resource availability can alter the distribution, abundance, demographic structure, reproduction, movement and aggregation of wildlife reservoir hosts. These changes may affect host density and contact patterns, influence pathogen maintenance and transmission within reservoir populations, and increase overlap with vectors, domestic animals and humans, thereby creating opportunities for zoonotic spillover [203]. In Australia, evidence is particularly strong for waterbirds and flying foxes, although climate-driven range shifts have also been documented in numerous mammalian hosts and arthropod vectors. At a global scale, climate and land-use change are projected to drive substantial redistribution of wildlife populations, increasing opportunities for novel host–pathogen interactions. By 2070, approximately 3870 mammalian species are predicted to experience geographic range shifts, resulting in twice the number of first-time species encounters compared with present-day conditions [7]. This reorganisation of mammalian communities may facilitate at least 15,000 novel cross-species viral transmission events between previously unexposed host species, highlighting the potential for climate change to accelerate viral emergence [7]. In wildlife reservoirs, these effects collectively drive shifts in host distribution, abundance, movement, aggregation, resource availability, and contact rates between species, although their relative importance varies among species and virus systems.

Among avian reservoirs, climate-driven changes in migration, breeding, and habitat availability are expected to alter the epidemiology of several important zoonotic viruses. Migratory bird populations are projected to shift poleward under warming climatic conditions, potentially modifying transmission pathways of HPAI H5N1 [204]. In Australia, however, the ecology of waterbird-associated viruses is strongly influenced by rainfall variability and extreme hydrological events. For example, MVEV activity in the south Kimberley region encompassing arid parts of south Western Australia has been linked to extensive rainfall and flooding both locally and in upstream catchments, which attract infected waterbirds from enzootic regions and create favourable conditions for viral amplification [205]. Thus, rainfall and flooding influence MVEV ecology by reshaping reservoir-host distribution and aggregation, with newly inundated wetlands attracting waterbirds and facilitating viral amplification and subsequent mosquito-mediated transmission. Temperature variation may further influence the dispersal of aquatic bird reservoirs, including herons that can harbour JEV without displaying clinical disease. Consequently, both daytime and night-time temperatures are important variables in models predicting the distribution of reservoir species and associated disease risk [206]. Together, MVEV and JEV illustrate a common climate-sensitive pathway in which rainfall, flooding, and temperature alter the waterbird distribution and aggregation and, consequently, opportunities for vector-mediated transmission.

The influence of climate variability on AIV ecology in Australia differs markedly from patterns observed in the Northern Hemisphere. Whereas migratory flyways largely determine influenza dynamics elsewhere, AIV prevalence in Australian waterfowl is strongly and positively associated with both long-term El Nino-Southern Oscillation (ENSO)-related wet conditions and rainfall during the preceding 3–7 months, indicating that ENSO indirectly influences AIV dynamics primarily through rainfall-driven ecological processes [201]. ENSO-related rainfall anomalies may increase temporary wetlands with greater food availability, the recruitment of larger numbers of immunologically naïve juveniles that may enhance host aggregation and contact rates and thereby facilitate AIV maintenance and transmission [201]. Because Australian waterfowl exhibit highly nomadic movement patterns in response to water availability and resource distribution, fluctuations in rainfall can substantially alter host aggregation, dispersal, and opportunities for virus spread [207]. Therefore, in Australian waterbirds, climate variability may influence AIV dynamics through at least three reservoir-mediated pathways: enhanced population recruitment, increased aggregation at resource-rich wetlands, and nomadic redistribution among wetlands. Temperature anomalies have also been associated with changes in seasonal abundance of key waterbird species, including the Chestnut Teal (*Anas castanea*), Australian Shelduck (*Tadorna tadornoides*), and Pacific Black Duck (*Anas superciliosa*), although their effects on AIV outbreaks appear complex and species-specific [201]. Thus, although AIV transmission differs from mosquito-borne MVEV and JEV transmission, all three systems demonstrate how climatic variability can restructure waterbird habitat, abundance, movement, and aggregation, thereby influencing viral maintenance and transmission.

The interaction between climate change and land-use modification may further elevate disease risks at the wildlife–livestock interface. Permanent water bodies, dams, and vegetated habitats on free-range farms can create refuges for nomadic waterfowl, increasing opportunities for sustained contact with domestic poultry. Consequently, exotic AIV incursions into Australian poultry systems are thought to occur more commonly through resident nomadic waterfowl that interact with migratory shorebirds at wetlands and coastal habitats, rather than through direct transmission from migratory birds to poultry [201]. Because migratory shorebirds rarely move inland, indirect transmission through local waterfowl populations is considered the more likely pathway [207]. Future climatic change may further alter migration timing, host distribution, and environmental persistence of viruses, potentially increasing the impact of HPAI on waterfowl populations [208]. Combined with the rapid evolution and global spread of influenza viruses, these climate-driven changes reinforce concerns about the potential introduction and establishment of HPAI in Australia and its potential implications for animal and human health [204,209,210].

While climatic influences on avian reservoirs primarily operate through changes in migration, breeding, and wetland availability, and resource distribution, similar processes are affecting mammalian reservoirs through habitat modification, food scarcity, and altered movement patterns. Flying foxes (*Pteropus* spp.) represent one of the best-studied examples of climate-mediated viral spillover in Australia. Habitat loss and deforestation may reduce the availability and distribution of native flowering forests that determine the nutrition and the traditional nomadic behaviour of flying fox populations, indirectly contributing to the emergence and spillover of HeV [211]. Changes in flowering forests availability may increase flying-fox movement and aggregation and alter their spatial and temporal overlap with horses, potentially affecting opportunities for HeV transmission. Evidence suggests that nutritional and environmental stress may influence flying-fox behaviour and virus dynamics; however, the relative contribution of climate-driven changes in food availability, host movement, and bat–horse contact to HeV spillover remains uncertain. This potential pathway is supported by a 25-year study in subtropical Australia where climate variability, habitat degradation, and reduced food availability have promoted a transition from seasonal migration to permanent residency in urban and agricultural landscapes [212]. Such changes in resource distribution and host movement may increase the frequency and duration of flying-fox aggregation in human-dominated environments and increase spatial overlap with horses, thereby creating more opportunities for HeV transmission at the wildlife–livestock interface [212].

Climate-related ecological stressors can further influence infection dynamics within flying fox populations. Food shortages associated with adverse climatic conditions can increase bat foraging activity in horse paddocks and agricultural areas, potentially increasing bat–horse contact and opportunities for HeV transmission, where horses act as amplifying hosts [212]. Similarly, extreme weather events, including cyclones, have been associated with elevated antibody prevalence in nutritionally stressed bat populations, suggesting enhanced viral circulation following ecological disturbance [213]. Seasonal patterns in HeV ecology are also evident, although infection, viral shedding, and spillover do not necessarily occur simultaneously. Infection prevalence in flying foxes often peaks during summer and autumn, whereas viral shedding and recorded spillover events are more frequently observed during winter [141,149,214]. Lower winter temperatures have been associated with elevated cortisol concentrations, a physiological stress response linked to increased HeV excretion [215,216].

Large-scale climate oscillations may also contribute to spillover risk. El Niño conditions, characterised by warmer and drier spring and summer periods, are associated with reduced food availability and increased dispersal of flying fox populations in search of resources [214,217,218,219,220]. These movements coincide with periods of elevated HeV prevalence and increased spillover risk during subsequent autumn and winter seasons [214,219,220]. Nevertheless, despite numerous ecological associations, few studies have demonstrated statistically significant relationships between spillover events and individual climatic variables such as temperature, rainfall, humidity, or ENSO indices [221]. This highlights the complexity of the ecological pathways linking climate change and disease emergence. This reservoir-mediated transmission pathway differs from the vector-mediated mechanisms observed for MVEV and JEV, but both illustrate a common principle whereby climatic conditions reshape host ecology and interactions at wildlife–vector or wildlife–domestic animal interfaces.

Importantly, climate change is expanding the geographic range of reservoir hosts and associated disease risk. The southward expansion of suitable habitat for the black flying fox (*P. alecto*) has increased the geographic extent of HeV spillover risk, resulting in an estimated 175–260% increase in the number of horses exposed to potential infection [203]. Overall, these findings suggest that climatic and environmental variability may influence HeV emergence through interconnected effects on resource availability, flying-fox movement and aggregation, physiological condition, viral circulation and shedding, and bat–horse contact, although the relative contribution of each pathway remains uncertain. More broadly, across wildlife-associated viral systems, climate change and environmental variability can modify the ecological processes that govern reservoir host distributions, migration patterns, reproductive dynamics, abundance, and interspecies interactions. By reshaping contact networks among wildlife, domestic animals, vectors, and humans, these climate-sensitive ecological changes can increase opportunities for viral emergence, persistence, and zoonotic spillover in Australia. Integrating climate and environmental monitoring with surveillance across wildlife, vector, domestic animals, and humans could therefore improve early identification of conditions favourable for viral emergence.

### 3.2. Climate Change Effects on Viral Transmission and Environmental Persistence

Climate change and extreme weather events can influence viral emergence by modifying the ecological conditions that support viral amplification, host–vector contact, viral evolution, and environmental persistence. In Australia, these effects are particularly evident for arboviruses, where temperature, rainfall, flooding, and hydrological variability regulate mosquito abundance, reservoir host dynamics, and transmission suitability. Rather than acting on viruses in isolation, climatic drivers create ecological contexts that favour viral maintenance, amplification, and, in some cases, the emergence of more pathogenic or better-adapted variants.

Rainfall is one of the most consistent environmental drivers of MVEV activity. MVEV transmission is strongly associated with above-average rainfall, flooding, and increased mosquito abundance, which together generate favourable conditions for *Orthoflavivirus* amplification and spread (Figure 2b) [106,184,190]. In Western Australia, monthly rainfall between 1990 and 2011 ranged from 0 to 608 mm in the Kimberley and 0–364 mm in the Pilbara. During this period, each 1 cm increase in monthly rainfall was associated with a 4.3–10% increase in sentinel chicken seroconversion rates at a two-month lag, with subsequent associations observed for both MVEV-positive chickens and human cases at a three-month lag [189]. Risk modelling further indicates that monthly precipitation below 40 mm is generally unsuitable for transmission, whereas suitability increases above 50 mm and becomes high beyond 75 mm in temperate Australian regions [196]. Temperature also modifies MVEV transmission potential, with mean maximum temperatures of 26–30.4 °C and mean minimum temperatures above 16 °C associated with increased suitability [12,188]. Notably, minimum temperature conditions were highly suitable during the 2021–2022 outbreak year [188].

Similar climate-sensitive patterns have been described for RRV, although the strength and direction of associations vary across regions, temperature metrics, and lag periods. In Townsville, Australia, rainfall, high tide events, and maximum temperature have been identified as key environmental drivers of RRV transmission [222]. RRV transmission suitability increases rapidly when rainfall exceeds average levels (Figure 2b). In Queensland, a Bayesian spatiotemporal model estimated that each 10 mm increase in monthly rainfall above a 50 mm baseline was associated with a 2.0% increase in average monthly RRV incidence [183]. Comparable rainfall thresholds have been reported elsewhere: in Alice Springs, transmission suitability increased rapidly when monthly rainfall exceeded approximately 100 mm during the December–March period [184], whereas in Broome, Western Australia, a monthly rainfall threshold of approximately 117 mm was associated with a 95% probability of an RRV outbreak [185]. These findings highlight rainfall as a critical determinant of mosquito habitat availability and epidemic potential.

Temperature effects on RRV transmission are also important but context-dependent. Monthly mean minimum and maximum temperatures, total rainfall, Southern Oscillation Index, and Murray River flow have been associated with increased likelihood of disease transmission, whereas higher relative humidity was associated with reduced transmission risk, with effects varying across time lags [223]. In southeastern Tasmania, a 1 °C increase in monthly mean maximum temperature within the range of 21–23.8 °C was projected to increase RRV cases by 23.2% above the long-term average at a one-month lag (Figure 2a) [224]. Other analyses have shown that increases in absolute monthly minimum temperature, monthly mean minimum temperature, and monthly mean temperature were each associated with increased RRV notifications at a one-month lag [225]. Together, these studies indicate that warming may expand transmission suitability in some regions, although local ecological and hydrological conditions remain critical modifiers.

BFV also shows strong climatic sensitivity, particularly to temperature and rainfall. Increasing minimum and maximum temperatures significantly increase BFV incidence and outbreak risk (Table 2 and Figure 2a). A 1 °C rise in minimum and maximum temperature has been associated with a 16.2% increase in transformed incidence at the current month and at two- to five-month lags [191], as well as a 14.9% increase in BFV incidence at lags of zero to two months [46]. In coastal Queensland, BFV risk modelling identified minimum temperature, rainfall, tidal conditions, and socioeconomic–environmental factors as key predictors of outbreak likelihood, with climate change scenarios shifting projected high-risk areas around Brisbane, Cairns, Mackay, Rockhampton, and the Gladstone–Gympie corridor [192]. Rainfall generally shows a positive association with BFV incidence (Figure 2b); for every 1 mm increase in annual average rainfall, the odds of a BFV outbreak increased by approximately 4% [192,193]. These findings indicate that BFV transmission is regulated by interacting climatic and environmental factors rather than by a single climatic variable.

Extreme rainfall and flooding can also create conditions for viral emergence and evolutionary change. During the 2010–2011 WNV/Kunjin outbreak in New South Wales, infection was largely confined to inland areas where flooding supported large mosquito populations and waterbird reservoirs. Genomic analysis of equine and mosquito isolates identified the WNV/Kunjin variant WNV_NSW2011, which was more neuroinvasive than the prototype Australian Kunjin strain and contained virulence-associated genetic changes [64]. This outbreak illustrates how climate-driven ecological amplification may coincide with the emergence or detection of viral variants with altered pathogenic potential.

Beyond effects on transmission intensity, climatic conditions may influence viral adaptation and genotype-specific fitness. Experimental evidence suggests that viruses can adapt to warmer environments and exhibit genotype-specific differences in persistence and transmission. For example, higher temperatures enhance the competitive advantage of the WN02 genotype of WNV over the NY99 genotype, with the advantage becoming more pronounced as temperature increases. Compared with NY99, WN02 is better adapted to warmer conditions, and the fraction of *Culex pipiens* mosquitoes capable of transmitting WNV increased more rapidly for WN02 than for NY99 [226]. JEV also shows genotype–climate associations. Genotype III and the recently emerged GI-b appear to be temperate-adapted and may be maintained year-round in northern latitudes, whereas GI-a and GII are more closely associated with tropical environments, where transmission is maintained through mosquito–bird and mosquito–swine cycles [227]. In tropical regions, JEV is endemic, and transmission can occur year-round at relatively low incidence [101,228]. Climatic anomalies associated with La Niña may further increase outbreak risk, with a 1 mm increase in average precipitation or a 1 °C cooler-than-normal temperature associated with approximately 38% and 22% increases in JEV outbreak risk, respectively [197].

Climate change and variability may also facilitate novel viral evolution by altering cross-species transmission opportunities. Global projections suggest that climate-driven redistribution of wildlife reservoir hosts will increase novel host contacts and may promote cross-species viral transmission [7]. At the wildlife–livestock interface, such processes are particularly relevant to HPAI H5N1, where interactions among migratory birds, resident waterfowl, and domestic poultry can create opportunities for viral reassortment, mutation, and selection of variants with enhanced transmissibility or pathogenicity [204,229]. AIV is especially prone to evolutionary change because of its segmented genome, high reassortment potential, and capacity for persistence in avian reservoirs. In the Southern Hemisphere, AIV dynamics differ markedly from the seasonal patterns observed in temperate Northern Hemisphere regions, reflecting distinct ecological, climatic, and host movement processes [124,230].

Environmental persistence is another key mechanism through which climate can shape viral transmission. For example, AIV is commonly transmitted through faeces-contaminated water, increased water temperature and salinity reduce viral survival time, thereby altering environmental persistence and transmission dynamics [231]. Unlike MBVs, rainfall affects AIV transmission primarily by expanding wetland habitats and increasing waterfowl abundance, rather than by directly influencing vector ecology. Wetter conditions may therefore increase opportunities for viral maintenance, reassortment, and spillover into domestic poultry by concentrating susceptible and infected birds in shared aquatic habitats. Respiratory viruses exhibit distinct climatic responses compared with arboviruses. In New South Wales, temperature was strongly associated with influenza risk, with relative risks ranging from 1.16 to 3.90. Increased risk occurred mainly at cold extremes below 15–18 °C, whereas risk was lowest at moderate temperatures of approximately 18–22 °C (Table 2) [200]. This relationship suggests a U-shaped temperature response, although evidence for high-temperature effects is less robust than for cold extremes, consistent with findings from other regions such as Hong Kong and Shanghai [200,232]. Humidity is also an important determinant of influenza transmission, but its effects vary by virus type and climatic region. Relative humidity has been associated with increased influenza activity, although associations with influenza B are less consistent. Absolute humidity appears to show stronger associations, with negative relationships reported for influenza B activity in temperate regions and positive associations with influenza A and B activity in subtropical and tropical regions [232].

However, Australian influenza dynamics cannot be explained by short-term climatic anomalies alone. Analysis of 15 years of city-level surveillance data found that anomalous fluctuations in temperature and humidity were not reliable predictors of local influenza epidemic onset timing [233]. Epidemics occurred under both high and low temperature and absolute humidity conditions, with no statistically significant associations observed in four of five cities studied, including Adelaide, Brisbane, Melbourne, and Perth [233]. These findings suggest that while climate influences influenza transmission, epidemic timing in Australia is also shaped by population immunity, virus importation, human behaviour, and other non-climatic factors.

Environmental conditions also regulate the persistence of bat-borne viruses such as HeV. HeV survival is consistently higher in long grass with low canopy openness than in short grass with high openness. Temperature-based survival estimates indicate that virus accumulation over successive days is greatest in long grass during winter, while evaporation further increases the contrast between long and short grass throughout the year [234]. Under conditions that promote rapid viral decay, transmission is likely to require direct or near-direct contact shortly after viral excretion. In contrast, when desiccation is low and viral survival is primarily temperature-dependent, HeV may remain viable for several hours in the environment, increasing the potential for indirect bat-to-horse transmission [138,235].

The available evidence indicates that temperature and rainfall interact to regulate the emergence, amplification, evolution, and persistence of climate-sensitive viruses in Australia. For arboviruses, temperature primarily influences mosquito physiology, viral replication, and vector competence, whereas rainfall determines breeding habitat availability, vector abundance, and amplification host dynamics. Integrated climatic response curves indicate that several Australian arboviruses have distinct thresholds beyond which transmission potential increases rapidly. As climate change intensifies warming, extreme rainfall, flooding, and hydrological variability, the spatial and seasonal suitability for RRV, BFV, MVEV, JEV, and WNV/KUNV may expand. In contrast, respiratory viruses such as AIV display different climatic responses, with cold and moderate humidity conditions often more important for transmission than warming alone. These divergent patterns highlight the need for virus-specific climate adaptation strategies within a One Health framework.

### 3.3. Climate Change and Variability Effects on Vector Biology, Distribution, and Vector-Borne Virus Transmission

Climate change and climate variability can influence vector-borne virus transmission by altering mosquito ecology and competence, vector distribution and abundance, host–vector contact, and temperature- and rainfall- dependent pathogen development or viral replication within vectors. In Australia, mosquito-borne disease transmission is governed by complex interactions among vectors, vertebrate reservoir hosts, amplifying hosts, and susceptible humans, all of which are influenced by temperature, rainfall, flooding, tides, and land-use change [236]. Across these systems, climatic influences predominantly converge on four interconnected pathways: vector development and competence, vector abundance and distribution, pathogen replication within vectors, and vector–host contact. Temperature and rainfall thresholds provide quantitative evidence that climate change can modify the seasonal and geographical suitability for arboviral transmission; however, such meteorological associations should not, in isolation, be interpreted as evidence of long-term climate-change effects. Where observed long-term climatic trends or climate projections coincide with changes in vector ecology or transmission suitability, they provide stronger evidence for potential climate-change effects, although the magnitude and direction of these effects differ substantially among viruses and ecological regions.

Temperature is one of the most important determinants of mosquito development and can also influence vector competence and pathogen development within mosquitoes. The thermal suitability curves for several Australian MBVs closely align with the developmental response of *Culex annulirostris*, an important Australian vector associated with Ross RRV, MVEV, BFV, KUNV, and JEV (Figure 2a). *C. annulirostris* exhibits a non-linear thermal response, with favourable development at 20–30 °C, greatest performance near 25 °C, and reduced performance at lower temperatures such as 15 °C; development may occur up to approximately 40 °C, with a minimum threshold around 17.5 °C (Figure 2a) [237]. More broadly, mosquito-borne disease transmission shows a non-linear thermal response, with peak suitability generally occurring between 23 °C and 29 °C and declining toward zero below lower thermal limits of 9–23 °C and above upper thermal limits of 32–38 °C [238,239]. For RRV, transmission potential also follows a unimodal thermal response, declining to zero at a lower limit of 17.0 °C and an upper limit of 31.5 °C, with peak estimated reproductive potential at 26.4 °C (Table 2 and Figure 2a) [182]. These threshold-based responses are critical for identifying periods and regions of increased outbreak risk under future warming scenarios. These temperature-dependent responses indicate that warming can alter the seasonal and geographical suitability for vector development; however, the implications for virus transmission depend additionally on mosquito survival, abundance, biting activity, vector competence, and host availability. Therefore, in Australian MBVs, temperature shapes transmission via several interconnected components of the vector–pathogen system, rather than through a straightforward, linear increase in disease risk with warming.

Mosquito biology represents one of the clearest mechanistic pathways linking climate change to arbovirus transmission in Australia. Temperature influences mosquito development time, larval survival, adult longevity, body size, blood-feeding behaviour, fecundity, and metabolic activity. It also affects viral replication within the mosquito and can shorten the extrinsic incubation period, thereby increasing the probability that mosquitoes survive long enough to become infectious before death [240,241]. Transmission is not possible when temperatures fall outside the limits required for mosquito survival, reproduction, and pathogen replication. Within suitable ranges, however, warming can increase mosquito activity, accelerate development, and enhance transmission potential [242,243]. These effects are highly non-linear, meaning that warming may increase transmission in cooler regions or seasons while reducing suitability where temperatures exceed upper thermal limits. Thus, the effects of warming on arbovirus transmission depend on the baseline thermal conditions, mosquito species, pathogen, and ecological context.

Rainfall and flooding primarily influence arbovirus transmission by regulating mosquito breeding habitat, vector abundance, and reservoir host aggregation. The 2011 MVEV outbreak provides a clear example: record wet-season rainfall and extensive flooding were associated with increased *Culex* mosquito abundance, sentinel chicken seroconversions, and human cases across multiple Australian jurisdictions [189]. MVEV may also persist through dry periods in desiccation-resistant eggs of *Aedes* species and remain dormant in arid environments until sufficient rainfall and flooding trigger renewed transmission. Isolation of MVEV from infected male *Aedes tremulus* mosquitoes supports the possibility of vertical transmission, although low-level localized transmission cannot be excluded [244]. These mechanisms suggest that extreme rainfall following prolonged dry periods may reactivate transmission cycles in areas where virus persistence is otherwise cryptic. In addition, long-term observations along the Murray River demonstrate that monthly rainfall exceeding 100 mm consistently precedes marked increases in mosquito abundance, with the prolonged high-rainfall and flooding events during 2021–2023 creating favourable conditions for amplified MVEV transmission (Figure 2b) [190]. Similar rainfall-driven increases in vector abundance and transmission have been documented for RRV and BFV, highlighting a recurring pathway from hydrological variability to mosquito habitat expansion, vector abundance, and subsequent human infection.

Rainfall-driven increases in mosquito abundance are also central to RRV and BFV transmission. Although rainfall, river height, and time-lagged mosquito abundance are significant predictors of RRV activity, their effects vary regionally [245,246]. Heavy rainfall and high tides can produce marked increases in *A. vigilax* and *C. annulirostris* abundance within two to four weeks. RRV notifications may then rise approximately eight weeks after rainfall events and remain above the previous five-year average for up to 21 weeks [187]. BFV notifications show a similar delayed pattern, increasing around nine weeks after heavy rainfall and remaining elevated for up to 23 weeks [187]. These lagged associations reflect the time required for rainfall to generate breeding habitat, increase adult mosquito populations, amplify infection, and produce detectable human cases. Such lagged responses demonstrate that climatic effects on disease operate through sequential ecological processes rather than occurring as immediate responses to weather conditions.

The relationship between rainfall and arbovirus transmission is nevertheless context dependent. In New South Wales, high rainfall following major bushfires substantially increased mosquito abundance and was associated with increased RRV activity [247]. However, the direction and strength of rainfall effects vary among ecological settings. For BFV, heavy rainfall has been associated with increased mosquito abundance and higher notifications in coastal areas [187]. In the Innisfail–Cassowary Coast region, average rainfall was positively associated with BFV notifications (Figure 2b), suggesting that rainfall promoted the formation and persistence of suitable breeding habitats [248]. Although a small negative rainfall effect was also reported, the magnitude was negligible, with each 1 mm increase in rainfall associated with only a 0.0003 decrease in the expected log count of BFV cases [248]. These findings suggest that rainfall can enhance transmission when it creates stable breeding habitats, but excessive rainfall may reduce transmission if it flushes larvae or disrupts breeding sites.

Temperature can also modify vector competence in virus- and mosquito-specific ways. Lower temperatures have been associated with higher infection rates in *Ochlerotatus vigilax* infected with RRV [249], whereas higher temperatures increased infection rates and shortened the extrinsic incubation period in *Culex* mosquitoes infected with WNV [250]. Similarly, Sindbis virus infection rates are higher in *Aedes aegypti* mosquitoes reared at 30 °C than in those reared at 20 °C, while lower rearing temperatures are associated with reduced susceptibility [251]. These findings indicate that rising temperatures may increase transmission potential for some vector–virus combinations but not universally, reinforcing the need for species-specific and virus-specific transmission models.

The recent emergence of JEV in southeastern Australia illustrates how climate anomalies can create novel overlap among vectors, reservoir hosts, amplifying hosts, and humans. JEV and WNV/KUNV share similar ecological pathways, with transmission commonly associated with above-average rainfall (Figure 2b) and La Niña conditions that expand temporary wetlands and waterways. Between 2020 and 2023, severe flooding events coincided with increased mosquito populations and arboviral disease activity, including the emergence of JEV in southeastern Australia [252]. The primary Australian JEV vector, *C. annulirostris*, is active across a temperature range of approximately 10.32–32.49 °C (Figure 2a) [194]. Experimental studies showed that at a mean temperature of 26.5 ± 1 °C, *C. annulirostris* exhibited high competence for the JEV genotype IV outbreak strain NSW/22, with 100% viral dissemination and 87% transmission [195]. These findings indicate that Australian mosquito populations are capable of efficiently transmitting outbreak-associated JEV strains under suitable climatic conditions.

The 2022 JEV outbreak was strongly associated with La Niña conditions that produced record rainfall and flooding across eastern Australia (Figure 2b). These conditions expanded mosquito breeding habitats and facilitated the movement of feral pigs, a key amplifying host, into new areas, thereby generating unprecedented virus–vector–host overlap in temperate regions previously considered at lower risk [253]. Annual enzootic activity may be expected in northern Australia, with occasional epizootic activity in eastern and southern regions during or following years of above-average rainfall. In addition, fluctuating diurnal temperatures may influence transmission in ways that are not captured by models based solely on mean temperature [240]. Temperature variability and rising sea levels in coastal urbanized regions may further alter the timing and duration of JEV transmission, particularly because several potential JEV vectors are salt tolerant [206]. Together, the JEV and WNV/KUNV systems demonstrate how rainfall, flooding, temperature, and hydrological conditions can synchronously alter vector abundance and vertebrate-host distribution, creating transient increases in vector–host overlap. These changes may weaken historical geographical boundaries and allow neurotropic Orthoflaviviruses to emerge in temperate zones during favourable climatic anomalies [254].

WNV/KUNV transmission is similarly influenced by climate-sensitive vector and reservoir dynamics. Ambient temperature and rainfall are fundamental drivers of mosquito abundance, WNV amplification, and infection dynamics in endemic areas [122,255]. In Australia, the Kunjin strain of WNV is enzootic in northern regions but exhibits episodic activity in southern regions, often following periods of heavy rainfall [115]. The 2011 WNV/Kunjin outbreak was likely driven by increased *C. annulirostris* abundance following extensive flooding in eastern Australia [64,256]. Evidence from the United States further indicates that both intrinsic factors, such as host immunity, and extrinsic factors, such as climate, predict West Nile neuroinvasive disease incidence, with immunity and drought emerging as strong predictors at national and state levels [257]. Although drought can reduce mosquito abundance, it may paradoxically increase WNV infection rates in some North American systems [258]. The thermal transmission range of *Culex quinquefasciatus*, an important WNV vector, extends from 19.0 °C to 31.8 °C, with peak transmission suitability at approximately 25.2 °C [198].

These climatic effects can alter the spatial and temporal overlap between vectors and vertebrate hosts, thereby modifying opportunities for pathogen acquisition and onward transmission. In Australian RRV systems, rainfall, temperature, and tidal conditions influence mosquito abundance and habitat availability, potentially changing the frequency and timing of vector–host encounters and, consequently, the seasonality and intensity of transmission [259]. When competent vectors feed on infected vertebrate hosts, they can acquire RRV and subsequently transmit the virus to susceptible hosts following viral replication within the vector. In southwestern Western Australia, RRV transmission potential increased with *Aedes camptorhynchus* abundance but decreased with western grey kangaroo abundance, highlighting the combined influence of vector and host communities on transmission dynamics [260]. More broadly, RRV is maintained through geographically and ecologically variable mosquito–vertebrate transmission cycles involving macropods and other vertebrate hosts, with *Ae. camptorhynchus*, *Ae. vigilax*, and *Culex annulirostris* contributing to transmission under different environmental conditions [111]. These findings indicate that RRV transmission is shaped by the convergence of climatic conditions, vector abundance, vertebrate host communities, and their spatial and temporal overlap, illustrating a mechanism shared across several MBV systems.

Overall, climate change and variability are expected to alter vector-borne virus transmission in Australia through interacting effects on vector development and competence, abundance and distribution, pathogen replication and vector-host contact. Warming may expand transmission into cooler regions and seasons where temperatures approach optimal ranges, while extreme heat may reduce suitability where upper thermal thresholds are exceeded. Increased rainfall, flooding, tides, and sea-level rise may expand mosquito breeding habitats, intensify vector abundance, and increase contact among mosquitoes, reservoir hosts, amplifying hosts, and humans. However, whether these mechanisms translate into increased disease emergence depends on mosquito species, viral genotype, local hydrology, land use, host ecology, and regional climate patterns. Effective prediction and control of MBVs will therefore require integrated surveillance systems that combine climate monitoring, mosquito ecology, wildlife and livestock surveillance, viral genomics, and human disease notification data within a One Health framework.

### 3.4. Climate Change and Variability Effects on Host–Pathogen–Vector Interfaces and Spillover Risk

Climate change and variability may increase viral spillover risk by reshaping the interfaces among pathogens, wildlife reservoirs, vectors, domestic animals, and humans rather than acting on viruses in isolation (Figure 1) [9]. These risks are spatially heterogeneous because climatic suitability interacts with host distribution, vector ecology, land use, and human and livestock exposure. An estimated 2.23 million Australians, or 8.7% of the population (95% CI: 73,186–9,120,601), may be at risk of JEV spillover based on ecological suitability and population density, with the highest proportions in New South Wales (11.9%), Queensland (8.5%), and Victoria (7.9%) [261]. Between 2001 and 2020, there were 43,699 reported cases of RRV in Queensland, with incidence markedly concentrated in the Warm climatic region (25,949 cases), followed by the Hot (13,422) and Dry (4325) regions, indicating significant spatial variation in RRV burden across the state’s climatic zones [262].

The effects of climate change and variability can converge on wildlife–vector interfaces by altering host and vector abundance, distribution and seasonal activity, thereby changing the timing and location of their overlap [263]. In Australian RRV systems, transmission involves multiple mosquito and vertebrate species, with higher transmission risk occurring where competent vectors, amplifying hosts, and favourable environmental conditions coincide [30]. For example, RRV hotspots in south-east Queensland occur at rural–urban interfaces where residential, agricultural, and conserved habitats bring mosquito, wildlife, and human communities into close proximity [263]. Although increased contact between infected hosts and competent vectors can facilitate viral acquisition and amplification, transmission remains dependent on the combined effects of vector activity, host availability, environmental conditions, and pathogen characteristics. Accordingly, temperature, rainfall, flooding, and tidal conditions may modify transmission suitability, but their effects are context-dependent and do not independently determine disease emergence [183].

For mosquito-borne Orthoflaviviruses such as MVEV and WNV/KUNV, heavy rainfall and flooding strengthen transmission cycles by expanding wetland habitats, increasing waterbird activity, and boosting *Culex* mosquito abundance. These conditions intensify the waterbird–mosquito–human or waterbird–mosquito–equine transmission interface (Figure 1) [64,109,188]. Together with RRV, these systems illustrate a common vector-mediated pathway in which climatic and hydrological conditions alter wildlife–vector overlap and consequently the probability of virus amplification and spillover. MVEV risk is strongly influenced by proximity to wetlands, although climatic variables and human population density also contribute to spatial variation in disease risk [188]. The identification of areas suitable for MVEV and JEV transmission during at least one summer month each year from 2016 to 2022, including both outbreak and non-outbreak years, suggests that environmental conditions favourable for MVEV and JEV transmission have persisted in parts of temperate Victoria beyond historically recognised outbreak years [188]. Although climatic suitability alone does not imply viral emergence or an outbreak, its persistence in temperate regions highlights the importance of considering climatic conditions alongside vector, host and ecological factors when assessing future transmission risk under climate change.

The emergence of JEV in Australia highlights the importance of climate-sensitive interfaces among birds, mosquitoes, pigs, and humans (Figure 1). The 2022 outbreak is consistent with a bird–mosquito–pig–human transmission cycle shaped by flooding, expanded mosquito habitat, and increased overlap between vectors and amplifying hosts. However, direct evidence linking specific climatic factors to the outbreak remains less established than for HeV, MVEV, or WNV/KUNV [264]. Nevertheless, the outbreak demonstrates how extreme rainfall and landscape-level ecological change can create novel transmission opportunities in regions previously considered at lower risk. Such events therefore illustrate how climatic anomalies can temporarily reorganise vector–host contact networks and create transmission opportunities beyond historically recognised risk areas and emphasize the importance of integrated surveillance across wildlife, livestock, vectors, and human populations.

Climate change also modifies mammalian reservoir interfaces, particularly for bat-borne viruses. The HeV system similarly illustrates how climate- and environment-driven changes in flying-fox distribution, food availability, and behaviour may modify bat–horse contact, although their contribution is difficult to disentangle from habitat loss, land-use change, and husbandry practices [215]. For HeV, 12 of 14 recorded spillover events between 1994 and 2010 occurred during the dry season, when rainfall, temperature, and vegetation productivity were consistently lower at affected sites [217]. These conditions may promote viral shedding in flying foxes, increase environmental persistence, or facilitate bat-to-horse transmission. Areas containing flying-fox roosts had substantially higher odds of HeV spillover than areas without roosts (OR = 40.5), suggesting that proximity to roosts may increase opportunities for bat–horse contact. Seasonal variation in climate and resource availability may alter flying-fox movements and host contact, potentially affecting the opportunities for Hendra virus spillover [217]. Unlike mosquito-borne systems, in which climate often acts through vector abundance and competence, HeV represents a reservoir-mediated pathway whereby climate and environmental conditions may alter resource availability, flying-fox movement, physiological condition, and bat–horse contact. Despite these differences, these findings support the concept that spillover risk is determined by the convergence of reservoir ecology, environmental stress, domestic animal exposure, and human land use rather than from any single driver.

Broader environmental change further increases the likelihood of zoonotic transmission by disrupting ecological and geographical boundaries. Urbanization, deforestation, intensified agriculture, and climate change have increased interactions among wildlife, livestock, and humans, creating ecological conditions that favour pathogen spillover [265]. These changes can force wildlife reservoirs into agricultural or peri-urban environments, increase reliance on anthropogenic food and water resources, and intensify contact with domestic animals. At the same time, climatic anomalies can alter vector breeding, host movement, and viral persistence, producing transient but high-risk windows for spillover.

Overall, these findings indicate that environmental and land-use changes can increase viral spillover risk by restructuring host–pathogen–vector interfaces rather than by uniformly increasing transmission of all viruses. The effects of these environmental changes vary among virus systems and depend on their specific reservoir, vector, and transmission ecology. For arboviruses, rainfall and flooding promote mosquito proliferation and reservoir host aggregation, strengthening waterbird–mosquito and livestock–human transmission pathways. For bat-borne viruses such as HeV, drought, food scarcity, habitat loss, and roost proximity can increase bat–horse–human contact and enhance opportunities for environmental or near-direct transmission. The convergence of climate extremes, land-use change, wildlife redistribution, and livestock exposure creates increasingly dynamic spillover landscapes.

## 4. Knowledge Gap and Recommendations

Wildlife-associated viral diseases represent an increasingly important One Health challenge in Australia, with implications for human, animal, and environmental health. Climate change and variability, urban expansion, land-use modification, and increasing interactions among wildlife, livestock, vectors, and humans are collectively reshaping the emergence, persistence, distribution, transmission, and spillover dynamics of these pathogens across tropical, subtropical, and temperate regions of Australia [266]. This review highlights the diverse roles of marsupials, wild birds, and bats in maintaining, amplifying, or facilitating transmission of several endemic and emerging viral pathogens, including MBVs such as RRV, BFV, JEV, MVEV, and WNV/KUNV, as well as bat-associated viruses including HeV, ABLV, and MenPV [17]. These wildlife hosts may also act as reservoirs or bridge hosts for introduced and emerging viral pathogens under changing environmental conditions.

Recent surveillance data indicate a concerning increase in mosquito-borne viral disease activity across Australia. National notifications of human MBVs infections nearly doubled between 2023 and 2024, with particularly large increases reported in Queensland, New South Wales, Victoria, and Western Australia. Queensland alone recorded 1701 RRV cases in 2024 [17]. Given the well-established wildlife–vector transmission cycles of many endemic arboviruses, these recent increases highlight the need to investigate the role of climate-sensitive ecological processes in shaping temporal and spatial variation in transmission.

This review was not meant to provide an exhaustive epidemiological evaluation of all wildlife-associated viral diseases, but rather a summary of critical overrepresented viral infections. Despite growing recognition of these risks, several critical knowledge gaps remain. First, comprehensive virome surveillance of Australian wildlife is still limited, particularly among marsupials, birds, and bats, restricting our understanding of viral diversity, host range, and spillover potential; this gap requires comprehensive wildlife surveillance, including metagenomic and metatranscriptomic approaches. Second, insufficient integration of wildlife surveillance, vector monitoring, climate data, and human health surveillance systems constrains prediction of spatial and temporal patterns of spillover, highlighting the need for spatially explicit and mechanistic modelling. Third, although climate variables such as temperature, rainfall, flooding, and ENSO cycles are known to influence transmission dynamics, the mechanistic pathways linking climate variability and long-term climate change to viral emergence and spillover remain poorly understood for many pathogens, requiring experimental and ecological studies to disentangle effects on reservoir physiology, vector competence, pathogen replication, movement, and host–vector or host–livestock contact. Finally, current surveillance and response frameworks remain fragmented across sectors, limiting the translation of ecological information into prevention, requiring an integrated One Health framework linking wildlife, veterinary, public health, environmental, and climate surveillance.

Addressing these gaps will require coordinated, multidisciplinary approaches that integrate wildlife health, veterinary science, public health, ecology, climatology, and genomics. Strengthening surveillance of wildlife reservoirs and mosquito vectors should be considered a cornerstone of efforts to prevent and mitigate the emergence of wildlife-origin viral diseases. Conventional surveillance remains essential for monitoring known pathogens, but it has limited capacity to detect novel, genetically divergent, or previously undescribed viruses in real time. Recent advances in metagenomic and meta-transcriptomic sequencing provide complementary tools for pathogen discovery and surveillance by enabling rapid detection and characterisation of known and emerging viruses directly from wildlife reservoir hosts and vector populations.

We recommend strengthening a nationally coordinated, climate-informed One Health surveillance framework linking Wildlife Health Australia, the Australian Centre for Disease Control, CSIRO’s Australian Centre for Disease Preparedness, the Australian Bureau of Meteorology, and relevant universities and research institutes. The framework should integrate: (i) routine surveillance of wildlife reservoirs (particularly marsupials, birds, and bats), and mosquito populations for early detection; (ii) genomic, experimental, and ecological studies to characterise viral diversity and clarify mechanisms of climate-sensitive transmission; (iii) integrated veterinary, wildlife, environmental, and public health surveillance to translate these findings into coordinated prevention and response. Combining these approaches with predictive disease-transmission models will strengthen early-warning systems, improve outbreak forecasting under future climate scenarios, support more timely and targeted public health interventions, and enhance Australia’s preparedness for emerging viral disease threats. Such an integrated framework will be critical for reducing the future burden of climate-sensitive wildlife-origin viral diseases and improving resilience to EIDs.

## 5. Conclusions

Climate change and climate variability are increasingly influencing the ecology and epidemiology of wildlife-origin viral diseases in Australia. Evidence synthesized in this review demonstrates that temperature, rainfall, flooding, and large-scale climate variability may interact to alter reservoir host distribution, vector abundance and competence, viral persistence, and opportunities for pathogen transmission and zoonotic spillover. These effects are particularly apparent for MBVs, including RRV, BFV, MVEV, JEV, and WNV/KUNV, while evidence for bat-associated viruses such as HeV indicates important but more complex links involving resource availability, host movement, physiological condition, and wildlife–livestock contact. Importantly, climate change and variability act primarily through ecological pathways, modifying interactions among wildlife reservoirs, vectors, livestock, and humans rather than directly affecting viruses alone. However, the extent to which short-term climate variability and extreme events translate into long-term changes attributable to climate change remains uncertain, and causal mechanisms are poorly resolved for many virus systems. The increasing frequency of extreme weather events, habitat alteration, and changing wildlife distributions may create novel transmission interfaces and expand opportunities for disease emergence. Together, the evidence highlights that understanding climate-sensitive host–pathogen–vector systems is essential for anticipating future disease risks in Australia. Australia should therefore prioritise integrated, climate-informed One Health surveillance linking wildlife, vectors, livestock, and human health; experimental studies to clarify climate-sensitive mechanisms; and spatially explicit predictive models to identify changing transmission windows and spillover hotspots. Integrating climate science with wildlife ecology, virology, and epidemiology will improve our ability to predict, detect, and respond to emerging viral threats in an increasingly dynamic environmental landscape.

## Figures and Tables

**Table 2:** Temperature and rainfall thresholds associated with relative transmission or outbreak suitability of selected One Health-related viruses in Australia.

Virus	Climatic Driver	Climatic Metric or Threshold	Direction of Effect on Cases	Key Impacts	References
RRV	Temperature	Thermal limits 17.0–31.5 °C, optimum 26.4 °C	Non-linear/Unimodal	RRV transmission declines to zero at a lower thermal limit of 17.0 °C and an upper thermal limit of 31.5 °C. Transmission potential R_0_ peaks at an optimal temperature of 26.4 °C.	[182]
	Rainfall	Monthly rainfall 14.15–216.83 mm, average monthly rainfall 50 mm, summer rainfall ˃100 to ˃400 mm	Increased	For 10 mm increase in monthly average rainfall 2.0% increase in the average monthly incidence of RRV. Mosquito abundance and RRV notifications increased.	[183,184,185,186,187]
MVEV	Temperature	Mean maximum temperatures 31.4 (30.4–33.0) °C mean optimum temperature 26.4 (26.2–26.8) °C, and mean minimum temperature 17.0 (16.0–19.2) °C.	Non-linear	MVEV dynamics change with maximum temperature. The mean minimum temperature was also highly suitable in the 2021–2022 outbreak year.	[12,188]
	Rainfall	Monthly rainfall 0–608 mm, summer rainfall ˃100 mm to ˃400 mm	Increased	For each 1 cm increase in monthly rainfall (from zero), 4.3–10% increase in chicken seroconversion rate at 2 months lag. Mosquito breeding habitats increased.	[106,184,189,190]
BFV	Temperature	Mean minimum temperature 18.75 °C, mean maximum temperature 27.44 °C, lowest maximum temperature 19.94 °C, highest maximum temperature 34.06 °C	Non-linear/Increased	BFV disease was statistically significantly associated with minimum temperature at the current month and at 2–5 months lags; for each 1 °C increase in minimum temperature a 16.2% increase in the transformed incidence rate. For each 1 °C increase in maximum temperature, there is a 14.9% increase in BFV incidence at lags of 0–2 months. Mosquito (*C. annulirostris*) development and viral replication increased.	[46,187,191]
	Rainfall	Mean rainfall 47.9 mm, Rainfall 10.9–269.1 mm	Positive	Positive significant association between rainfall and BFV incidence rate. For every 1 mm increase, a 4% increase in the odds of a BFV outbreak.	[192,193]
JEV	Temperature	Minimum temperature: 10.32 °C, Maximum 32.49 °C, mean temperature 26.5 °C ± 1 °C	Non-linear/Positive	*Culex annulirostris*, the primary JEV vector in Australia. At 26.5 °C ± 1 °C, *Culex annulirostris* shows 100% dissemination and an 87% positivity rate for JEV genotype IV outbreak strain (NSW/22).	[194,195]
	Rainfall/Preci-pitation	Unsuitable: 0–40 mm, increasing risk: 40–50 mm, medium risk: 50–75 mm, highest risk: >75 mm and above-average rainfall (Average: 40 mm)	Increased	Rise in JEV cases and transmission suitability increases	[194,196,197]
WNV	Temperature	Minimum temperature 19 °C, Optimum temperature 25.2 °C, maximum temperature 31.8 °C	Non-linear	*Culex quinquefascsiatus* development	[198]
	Rainfall	Above average rainfall	Increased	Waterbirds, mosquitoes, virus evolution	[64,67,116,199]
IAV	Temperature	Temperature below 15–18 °C (cold extremes), moderate temperature (18–22 °C)	Non-linear/Decreased	From 6.73 °C for each 1 °C increase, approximately a 20% decrease in influenza notifications. The temperature effects consistently show U-shaped relationships, but the data for high-temperature effects are significantly less robust than the data for cold extremes. Minimum risk for IAV at moderate temperatures.	[200]
	Rainfall	Higher rainfall	Increased	Following higher rainfall, waterbird numbers may increase in new temporary wetlands with greater food availability, which can support breeding and the recruitment of immunologically naïve juvenile birds at temporary wetlands. The subsequent movement of waterbirds between temporary and permanent water bodies may contribute to the maintenance and potentially increase AIV prevalence in wild waterfowl in Australia.	[72,201]

## Data Availability

No new data were created or analyzed in this study. Data sharing is not applicable to this article.

## Supplementary Materials

The following supporting information can be downloaded at https://www.mdpi.com/article/10.3390/v18091001/s1, Table S1: Temperature and rainfall data.
